# Attentional Modulation of Envelope-Following Responses at Lower (93–109 Hz) but Not Higher (217–233 Hz) Modulation Rates

**DOI:** 10.1007/s10162-017-0641-9

**Published:** 2017-10-02

**Authors:** Emma Holmes, David W. Purcell, Robert P. Carlyon, Hedwig E. Gockel, Ingrid S. Johnsrude

**Affiliations:** 1 Brain and Mind Institute, University of Western Ontario, Natural Sciences Centre, Room 120, London, ON N6A 5B7 Canada; 2 School of Communication Sciences and Disorders, University of Western Ontario, London, ON N6G 1H1 Canada; 3 MRC-Cognition and Brain Sciences Unit, University of Cambridge, Cambridge, CB2 7EF UK

**Keywords:** attention, FFR, EFR, EEG, brainstem

## Abstract

Directing attention to sounds of different frequencies allows listeners to perceive a sound of interest, like a talker, in a mixture. Whether cortically generated frequency-specific attention affects responses as low as the auditory brainstem is currently unclear. Participants attended to either a high- or low-frequency tone stream, which was presented simultaneously and tagged with different amplitude modulation (AM) rates. In a replication design, we showed that envelope-following responses (EFRs) were modulated by attention only when the stimulus AM rate was slow enough for the auditory cortex to track—and not for stimuli with faster AM rates, which are thought to reflect ‘purer’ brainstem sources. Thus, we found no evidence of frequency-specific attentional modulation that can be confidently attributed to brainstem generators. The results demonstrate that different neural populations contribute to EFRs at higher and lower rates, compatible with cortical contributions at lower rates. The results further demonstrate that stimulus AM rate can alter conclusions of EFR studies.

## Introduction

Understanding spoken language in the presence of other background sounds requires listeners to direct attention flexibly to distinguishing acoustic characteristics (e.g. the fundamental frequency of someone’s voice), an ability likely underpinned by dynamic interactions between basic auditory and higher-level cognitive processes (Carlyon et al. 2001; Davis and Johnsrude 2007; Billig et al. 2013). However, whether directing attention to particular sound frequencies alters processing at the earliest stages of the auditory system is unclear. Improving knowledge of how attention changes the representation of sounds at different stages of auditory processing is fundamental to understanding how listeners hear a sound of interest among a mixture of competing sounds.

Directing attention to sounds at different spatial locations affects cortical activity recorded using electroencephalography (EEG) (Hillyard et al. 1973; Parasuraman et al. 1982; Woldorff et al. 1987; Anourova et al. 2001; Bharadwaj et al. 2014), magnetoencephalography (MEG) (Woldorff et al. 1993; Xiang et al. 2010; Ding and Simon 2012), and functional magnetic resonance imaging (fMRI) (Petkov et al. 2004; Voisin et al. 2006; Formisano et al. 2008). Additionally, fMRI studies have demonstrated that auditory cortex activity is modulated by frequency-specific attention (Paltoglou et al. 2009; Da Costa et al. 2013; Riecke et al. 2016). However, whether top-down projections enable filtering of responses at lower stages of the auditory pathway, potentially facilitating perceptual segregation of sounds of different frequencies, is unclear. Descending anatomical projections from the cortex to the cochlea are, at least broadly, organised by frequency (Winer and Schreiner 2005), so it is anatomically plausible that attending to particular frequencies may enhance tuning or gain in a frequency-specific fashion at the earliest levels of auditory processing. Ongoing tuning of brainstem processing based on expectations and goals would permit auditory processing to rapidly adapt to changes in listening environment and would allow listeners to flexibly enhance processing of target sounds.

The envelope-following response (EFR) is a steady-state electrophysiological response that tracks periodic features of the amplitude envelope of a stimulating sound. EFRs differ depending on whether participants direct attention to auditory or visual stimuli (Galbraith and Arroyo 1993; Hoormann et al. 2000; Galbraith et al. 2003), but results are inconsistent. When participants direct attention away from auditory stimuli (towards visual stimuli), some have observed a decrease in EFR amplitudes with no effect on latencies (Galbraith and Arroyo 1993; Galbraith et al. 2003), whereas others report an increase in latencies with no effect on amplitudes (Hoormann et al. 2000). Studies measuring frequency-following responses when attention is directed to different sounds are also inconsistent: frequency-following responses to the temporal fine structure of sounds were modulated by attention in one study (Galbraith and Doan 1995), but not in two others (Lehmann and Schönwiesner 2014; Varghese et al. 2015). Previous experiments vary in several ways, including the type of acoustic stimulus presented, the frequency of the stimulus eliciting EFRs, and the signal-to-noise ratio of measured EFRs, which may explain the inconsistencies.

EFRs are most commonly recorded using EEG. EFRs elicited by stimuli that have amplitude modulation rates of 70–200 Hz are commonly assumed to reflect neural activity within the rostral brainstem. This assumption is based on electrophysiological recordings (Worden and Marsh 1968; Marsh et al. 1974; Smith et al. 1975), measurements of group delay (Kiren et al. 1994; Herdman et al. 2002; King et al. 2016), and the long-standing belief that auditory cortex frequency-following drops off above about 50–70 Hz (implying that phase-locking at 70 Hz and higher must originate subcortically). However, studies using electrocorticography (ECoG)—an intracranial method that is sensitive to local electrical activity likely not present in EEG (Buzsáki et al. 2012)—have shown that the auditory cortex is capable of tracking frequencies up to 200 Hz (Brugge et al. 2009; Nourski et al. 2013; Behroozmand et al. 2016). In addition, two recent studies using MEG (Coffey et al. 2016) and EEG (Coffey et al. 2017) indicate that cortical generators likely contribute to frequency-following activity at 98 Hz. Nevertheless, it remains unclear whether such cortical contributions are sufficiently large, relative to brainstem generators, to influence the outcomes of EEG studies measuring EFRs at 70–200 Hz.

We recorded EFRs at two sets of modulation frequencies—one within the range traditionally used for EFR recordings (70–200 Hz; experiment 1) and one at higher rates that only brainstem but not cortex is able to track (> 200 Hz; experiment 2). Using EEG, we compared EFRs when participants attended to concurrently presented tone streams of different frequencies. If frequency-specific attention modulates brainstem processing, then EFRs should be modulated by frequency-specific attention at both sets of modulation rates.

## Methods

### Participants

Participants in experiment 1 were 30 right-handed young adults. Experiment 1 included two separate versions of the attend-auditory task (as described below). Pre-established criteria for excluding participants included audiometric thresholds outside of the normal hearing range or poor task performance (negative d′ for the auditory or visual detection tasks). We excluded six participants due to poor auditory task performance. The remaining 24 participants (12 male) were aged 18–27 years (mean [*x̅*] *=* 20.5, standard deviation [*s*] = 2.7). Participants had average pure-tone hearing levels of 20 dB HL or better (at six octave frequencies between 0.5 and 8 kHz).

Participants in experiment 2 were 14 right-handed young adults. We excluded one participant due to poor auditory task performance and one due to high audiometric thresholds in the left ear at 4 and 8 kHz. The remaining 12 participants (4 male) were aged 19–26 years ($\overline{\mathrm{X}}$ *=* 22.3, *s* = 2.2) and had average pure-tone hearing levels of 20 dB HL or better (at six octave frequencies between 0.5 and 8 kHz).

Both experiments were cleared by Western University’s Health Sciences Research Ethics Board. Informed consent was obtained from all participants.

### Apparatus

The experiments were conducted in a sound-insulated and electromagnetically shielded double-walled test booth (Eckoustic model C-26 R.F.). Participants sat in a comfortable chair facing a 22-in. visual display unit (ViewSonic VS2263SMHL).

Acoustic stimuli were presented through a LynxTWO-A sound card (Lynx Studio Technology, Inc.). Stimuli were delivered binaurally through Intelligent Hearing Systems mu-metal shielded Etymotic Research ER2 earphones, which were clipped to the chair and sealed in the ear canal of the listener with disposable foam inserts.

### Stimuli

#### Acoustic Stimuli

Acoustic stimuli for both experiments were three simultaneous streams of tones at three perceptually distinct carrier frequencies (1027, 1343, and 2913 Hz in Experiment 1, and 1753, 2257, and 4537 Hz in Experiment 2) that we trained the listeners to think of as ‘low’, ‘middle’, and ‘high’ frequencies. Each tone stream was ‘tagged’ with a unique AM rate, so that we could isolate the EFR to each stream separately. In Experiment 1, the AM rates for the low-, middle-, and high-frequency streams were 93, 99, and 109 Hz, respectively, whereas in Experiment 2, they were 217, 223, and 233 Hz.

**Fig. 1:**
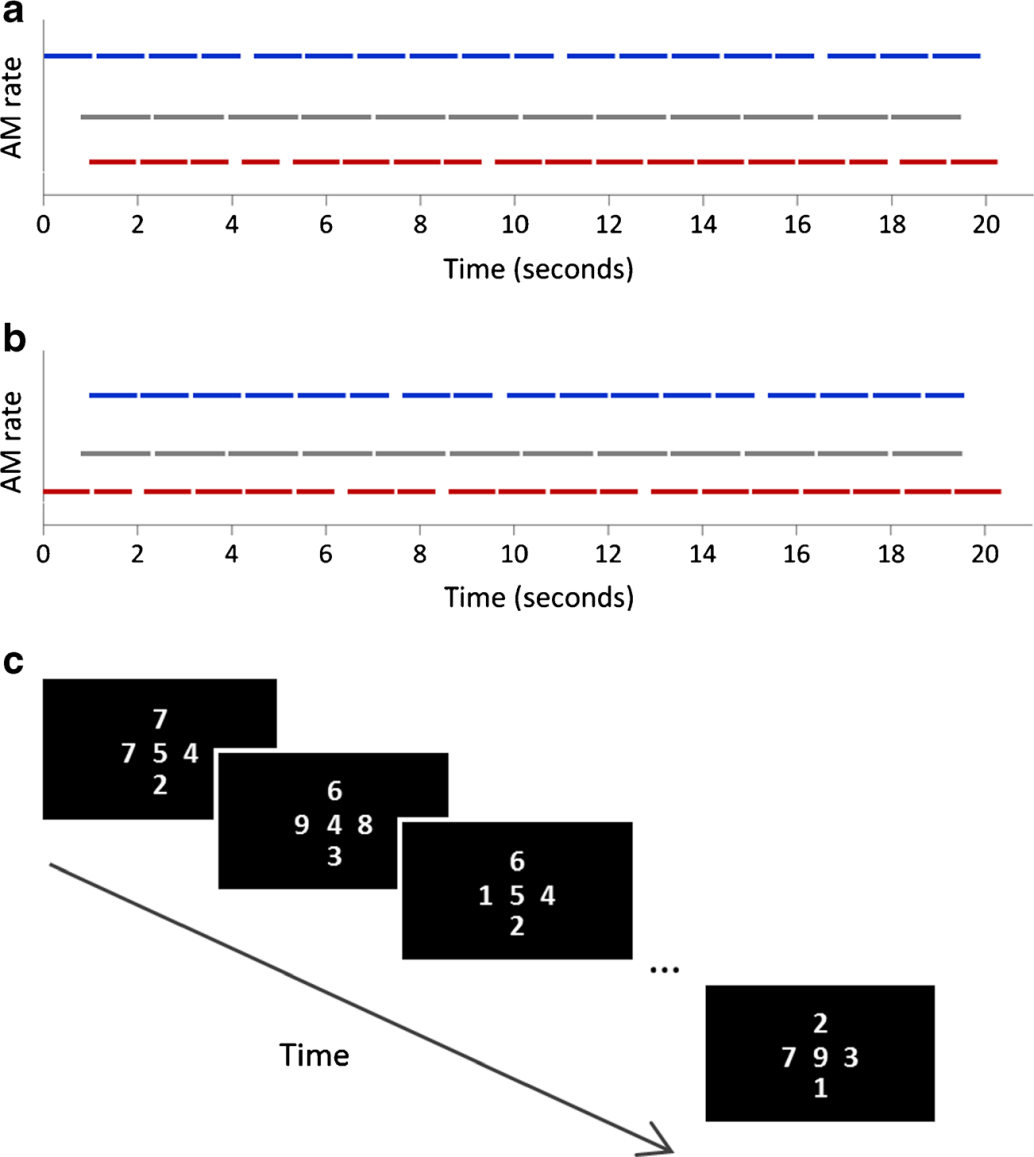
**a** Schematic of tone onset times for trials in which the high-frequency stream began first. Each colour represents a stream of tones that have different frequency carriers and are tagged with different AM rates. The tones in the three different streams also had three different durations and unique inter-stimulus intervals. Shorter deviant stimuli occurred in the high- and low-frequency streams. **b** Same for trials in which the low-frequency stream began first. **c** Schematic of visual stimuli

To promote perceptual segregation, tones in the three different streams also had three different durations (1036, 1517, and 1052 ms, for the low-, middle-, and high-frequency streams, respectively) and unique inter-stimulus intervals (51, 63, and 71 ms, respectively), so that the onsets of tones from the three streams occurred at different times (see Fig. 1a). All tones had cosine onset ramps of 10 ms and were sampled at 32,000 samples/s. The level of each stream was set to 70 phons, according to the ISO 226 normal equal-loudness-level contours (ISO 226 2003). On half the trials, the polarity of the temporal fine structure was inverted, so that averaging responses across stimuli would emphasise the envelope response and cancel any artefact related to the stimulus temporal fine structure (Picton and John 2004; Small and Stapells 2004).

On each trial, either the high- or the low-frequency stream started first (compare Fig. 1a, b), which cued the listener to attend to that stream to perform the detection task in the Attend-Auditory condition that is described below. The other two streams started 700 and 1000 ms (in a randomly determined order) after the onset of the first tone from the first stream. The low-, middle-, and high-frequency streams contained 18, 12, and 17 tones, respectively, except that the stream that started first also contained an additional tone, so that the streams ended at approximately the same time. Overall, the acoustic stimulus for each trial lasted approximately 21 s.

During the main parts of the experiment, participants performed a deviant-detection task on whichever stream they were instructed to selectively attend. Both the high- and the low-frequency streams contained three to four shorter deviant tones on every trial. The middle-frequency stream was never the target stream and did not contain deviant stimuli; the purpose of the middle-frequency stream was to make the auditory task more difficult. The durations of shorter deviants depended on each participant’s deviant-detection threshold, which was determined during a preliminary phase of the experiment, described below.

#### Visual Stimuli

The visual stimulus on each trial consisted of five digits selected from the numbers 1–9. One digit was presented in the centre of the screen and four digits were presented above, below, to the left, and to the right of the central digit, as illustrated in Fig. 1c. The digits were selected with replacement. A new array of digits was presented every 750 ms, lasting throughout the full duration of the acoustic stimulus for each trial (28 arrays of digits per trial).

### Procedures

For both experiments, participants were first trained to perform the tasks. They first heard examples of the high- and low-frequency tone streams alone (three tones per example). Participants were allowed to listen to the examples as many times as they liked. Next, participants completed 16 training trials, in which they heard shorter extracts (~ 4-s duration) of the acoustic stimuli used in the main experiment. For these stimuli, participants were instructed to attend to either the highest- or the lowest-frequency tone stream in separate blocks. On each trial, either 0 or 1 deviant stimulus (shorter in duration than the standard) was present in the high- and low-frequency streams. During the first half of practice trials for each attended frequency, deviant tones were 30 % shorter than the standard tones in the stream. During the second half of practice trials at each attended frequency, deviants were 10 % shorter. Participants performed a two-alternative forced-choice task, in which they had to detect whether or not the attended stream contained a shorter deviant tone. Visual feedback was provided during training.

After training, the durations of the shorter tones were altered in an adaptive procedure until the hit rate was between 70 and 85 %. The acoustic stimuli had the same structure as in the main part of the experiment. Participants were instructed to attend to the stream that began first and press a button as quickly as they could whenever they detected deviants in the attended stream, while ignoring deviants in the other streams. The durations of shorter deviants in the high- and low-frequency streams were adapted in separate, but interleaved, runs.

The main part of each experiment comprised three different blocks: Attend-Auditory, Attend-Visual, and Artefact Check. The order of the three blocks was counterbalanced across participants.

#### Attend-Auditory Condition

In the Attend-Auditory condition, participants had to detect shorter tones within either the high- or low-frequency tone stream—whichever began first (see Fig. 1a, b). Participants had to press a button as quickly as they could whenever they detected a shorter deviant in the target stream, while ignoring deviants in the other streams. There were 120 trials in the Attend-Auditory condition (60 Attend-High and 60 Attend-Low). Attend-High and Attend-Low trials were randomly interleaved within each block.

In Experiment 1, 12 participants saw a visual fixation cross throughout the Attend-Auditory condition, and the changing digit array only when performing the Attend-Visual task. Thus, for these participants, the acoustic stimuli were identical across attentional conditions (Auditory and Visual), but the visual stimuli differed. The other 12 participants in Experiment 1 and all the participants in Experiment 2 saw the changing digit array in the Attend-Auditory condition. Thus, for these participants, both the acoustic and visual stimuli were identical in the Attend-Auditory and Attend-Visual conditions. All participants were instructed to fixate on the centre of the screen but focus their attention on the acoustic stimuli. Given there was no difference in EFRs between the two groups in Experiment 1 who experienced different visual stimuli during the Attend-Auditory condition, we analysed the data from all participants in Experiment 1 together.

#### Attend-Visual Condition

Acoustic stimuli in the Attend-Visual condition were identical to those presented in the Attend-Auditory condition—there were two different types of acoustic stimuli, corresponding to the Attend-High and Attend-Low tasks, with either the high- or low-frequency tone stream starting first (see Fig. 1a, b). In the Attend-Visual condition, however, participants were instructed to ignore the acoustic stimuli and attend to the visual stimuli (Fig. 1c).

In the Attend-Visual condition, participants performed a two-back task on the central digit, while ignoring the four distracting digits. Participants had to press a button as quickly as they could whenever the central digit matched the central digit presented two arrays earlier. There were two to five visual targets per trial. There were 60 trials in the Attend-Visual condition (30 in which the high-frequency tone stream began first and 30 in which the low-frequency stream began first; the trial types were randomly interleaved).

We designed the visual task to be difficult so that participants would not be able to perform the task with high accuracy unless they were attending to the visual (rather than the acoustic) stimuli. The visual task had high perceptual load (four distracting digits and a relatively short inter-digit interval). Given that tasks with high perceptual load have been shown to reduce processing of distractor stimuli compared to tasks with low perceptual load (Lavie and Tsal 1994; Lavie 1995), we assumed that using a high-load visual task would minimise processing of acoustic stimuli in the Attend-Visual condition.

#### Artefact Check Condition

In the Artefact Check condition, acoustic stimuli were identical to the Attend-Auditory and Attend-Visual conditions. However, the foam inserts were taken out of the participant’s ears and covered with tape, so that the acoustic stimuli were still delivered to the earphones, but were not audible to the participant. In the Artefact Check condition, participants passively watched a subtitled DVD. There were 60 trials in the Artefact check condition (30 in which the high-frequency stream began first and 30 in which the low-frequency stream began first; the trial types were randomly interleaved).

### Behavioural Analyses

We calculated d′ (Green and Swets 1966) for the Attend-Auditory and Attend-Visual conditions. False alarms were defined as responses to non-deviant tones in the target stream. We used two-tailed paired-sample *t* tests to compare d′ between the auditory and visual tasks and, within the Attend-Auditory condition, between conditions in which participants attended to the high- or low-frequency stream.

### EEG Recording and Pre-processing

We recorded EEG using disposable Medi-Trace Ag/AgCl electrodes. The recording electrode was placed at the vertex (Cz), with a reference at the posterior midline of the neck (just below the hairline) and a ground (or common) on the left collarbone. Electrode impedances were below 5 kΩ at 10 Hz, and inter-electrode differences in impedance were less than 2 kΩ (measured using an F-EZM5 GRASS impedance meter). A GRASS LP511 EEG amplifier applied a gain of 50,000 with bandpass filtering at 0.3–3000 Hz. A National Instruments (Austin, TX) PCI-6289 M-series acquisition card captured the EEG data at a rate of 32,000 samples/s with 18-bit resolution. The PCI-6289 card applied a further gain of 2 for a total gain of 100,000. The recording program was custom developed using LabVIEW (Version 8.5; National Instruments, Austin, TX).

### EFR Analyses and Statistics

The EEG data were exported to MATLAB (version 2014b; The MathWorks, Inc., Natick, MA, USA) and were analysed using custom-written scripts. First, we isolated epochs corresponding to the times of tones in the high- and low-frequency streams. We ignored the first tone in each stream, then extracted epochs with 1-s duration at the beginning of the next 16 tones in each stream.

We used the Fourier transform (FT) to estimate the frequency spectrum of the response for each epoch, with the purpose of excluding noisy epochs. For each epoch, we averaged amplitudes at 80–200 Hz (excluding the frequencies of interest). We then calculated the mean and standard deviation across epochs for each participant within each condition and excluded epochs with amplitudes > 2 standard deviations from the mean. This led to the rejection of 2.7 % of epochs, on average, per participant in each condition. We computed the time-domain average of all remaining epochs. We averaged across epochs with opposite stimulus polarity so as to isolate the envelope response.

We computed the FT of the time-domain average to estimate the amplitude of the EFR at the AM rates of the low- and high-frequency streams (Experiment 1: 93 and 109 Hz; Experiment 2: 217 and 233 Hz); we refer to the two EFR frequencies of interest as the low and high EFR components. We also estimated the noise floor at each EFR component by averaging the amplitudes at the 10 adjacent frequency bands (five on each side; resolution 1 Hz). We calculated signal-to-noise ratios (SNRs) for each EFR component by dividing the EFR amplitude at the stimulus AM rate by the estimate of the noise floor at the adjacent frequencies.

The Attend-Auditory condition contained twice as many trials as the Attend-Visual and Artefact Check conditions; thus, in order to ensure that effects across conditions were estimated from the same quantity of data, we resampled half the total number of epochs (i.e. n/2) in the Attend-Auditory condition. We drew 500 samples of n/2 trials with replacement, computed the time-domain average within each sample, and then calculated the average EFR SNR, amplitude, and noise estimate across samples.

To analyse the effect of frequency-specific attention on EFRs, we focused on the Attend-Auditory and Attend-Visual conditions only. In the Attend-Auditory condition, participants were instructed to attend to the stream (high or low) that started first. Given the acoustic stimuli differed between these two types of trials (due to the earlier onset), we used the Attend-Visual condition as a baseline to control for possible stimulus-driven differences in EFRs. To that end, all conditions were split into trials in which the high- or low-frequency tone stream began first. We expected frequency-specific attention effects on the EFR in the Attend-Auditory condition but not in the Attend-Visual condition, in which the first tone stream had no implications for participants’ task. In contrast, stimulus-driven earlier onset effects (if present at all) would occur in both Attend-Visual and Attend-Auditory conditions. Importantly, the analyses compared trials (between auditory and visual attention) in which the acoustical stimuli were identical; this was done to extract the effect of frequency-specific attention from the physical stimulus differences. We used two-tailed within-subject ANOVAs to compare EFR SNRs across conditions (Attend-Auditory and Attend-Visual), stimulus types (high- or low-frequency tone stream beginning first), and EFR components. We used a combination of box plots, Q-Q plots, and the Kolmogorov-Smirnov test to check that the data did not deviate strongly from a normal distribution and we checked that the data met the assumption of sphericity.

To investigate whether the extent of EFR modulation by frequency-specific attention was related to task performance, we aimed to extract a measure of attentional modulation to correlate with performance on Attend-Low and Attend-High trials. For the low EFR component, we expected greater SNRs when participants attended to the low-frequency stream than when they attended to the high-frequency stream. Because these two attentional conditions also differed in the frequency of the first tone, we divided the ratio of the Attend-Low and Attend-High SNRs by the ratio of the SNRs in the corresponding Attend-Visual conditions (low-stream first vs. high stream first); the final measure was (Attend-Low / Attend-High) / (Attend-Visual (low stream first) / Attend-Visual (high stream first)). We expected the opposite pattern at the high EFR component—greater SNRs for Attend-High than Attend-Low trials. Thus, we inverted the ratios, consistent with the expected direction of modulation [i.e. (Attend-High / Attend-Low) / (Attend-Visual (high stream first) / Attend-Visual (low stream first))]. We also calculated Pearson’s product-moment correlations between Attend-Low d′ and the extent of EFR modulation at the low EFR component and also between Attend-High d′ and the extent of EFR modulation at the high EFR component.

In addition, we calculated phase coherence (Jerger et al. 1986; Stapells et al. 1987) at each EFR component, separately for each condition. Phase angles at each EFR component were analysed with the FT, then phase coherence was calculated as the root mean square of the sums of the cosines and sines of the individual phase angles. Similar to the other EFR measures, we used within-subject ANOVAs to compare phase coherence across conditions, stimulus types, and EFR components. We also calculated the extent of attentional modulation of EFR phase coherence using the same ratios as those described for SNR. We calculated Pearson’s product-moment correlations between Attend-Low d′ and the extent of EFR modulation at the low EFR component and between Attend-High d′ and the extent of EFR modulation at the high EFR component.

## Results

### Experiment 1: Low AM Rates

#### Task Performance

**Fig. 2:**
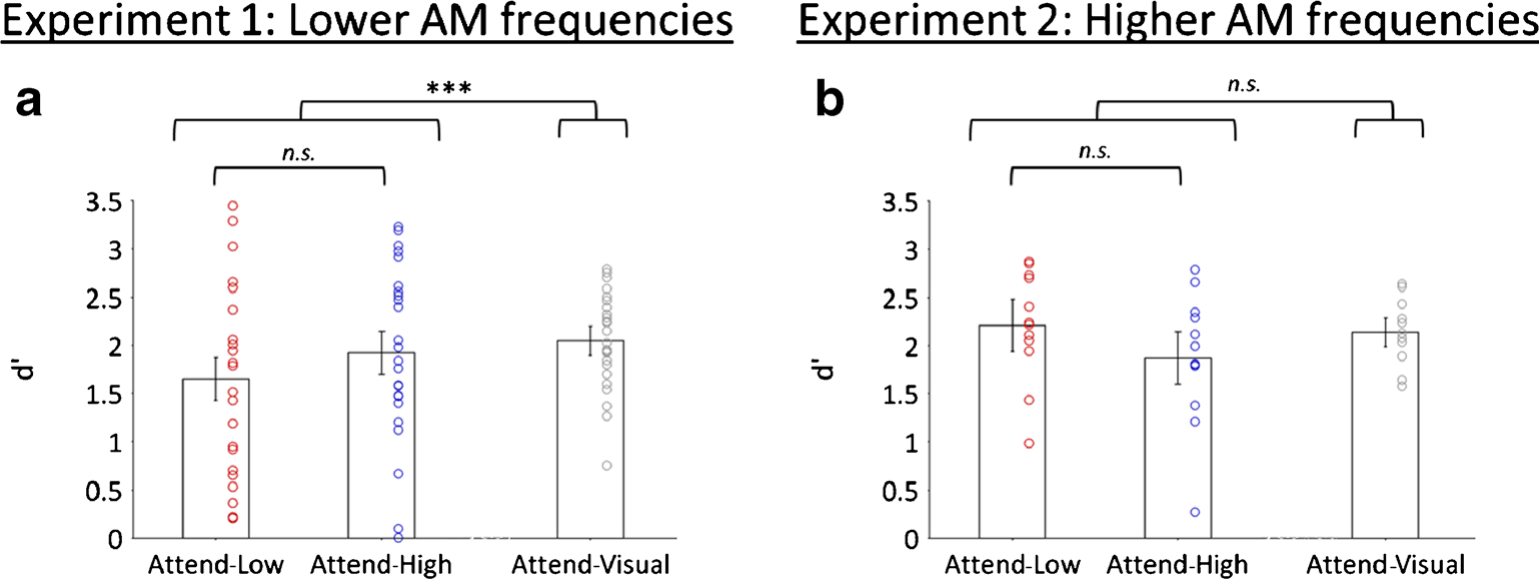
**a** Sensitivity (d*′*) for the Attend-Auditory and Attend-Visual tasks in Experiment 1 (*N* = 24). Error bars show within-subject 95 % confidence intervals. Circles display the results from individual participants. Brackets display the results from pairwise comparisons: *n.s.* not significant; *p < 0.05; **p < 0.01; ***p < 0.001. **b** Same for Experiment 2 (*N* = 12). *AM* amplitude modulation

Sensitivity (d′) varied substantially among participants in both the auditory (range 0.1–3.2; based on all trials, irrespective of attention condition) and visual (range 0.8–2.8) tasks. Participants performed significantly better on the visual task (*x̅ =* 2.1, *s* = 0.5) than the auditory task (*x̅ =* 1.5, *s*σ = 0.9) [*t*(23) = 3.69, *p* = 0.001]. Figure 2a illustrates d′ for the auditory (separated into Attend-Low and Attend-High trials) and visual tasks. Within the auditory task, performance did not differ significantly between Attend-High (*x̅ =* 1.6, *s* = 0.9) and Attend-Low (*x̅* = 1.4, *s* = 1.0) trials [*t*(23) = 1.31, *p* = 0.20]. Participants did not frequently respond to deviants in the non-target stream (Attend-Low: *x̅* = 3.0 % of non-target deviants, *s* = 2.9; Attend-High: *x̅ =* 1.3 %, *s* = 1.6).

#### Comparison of EFRs Between Conditions and Spectral Components

**Fig. 3:**
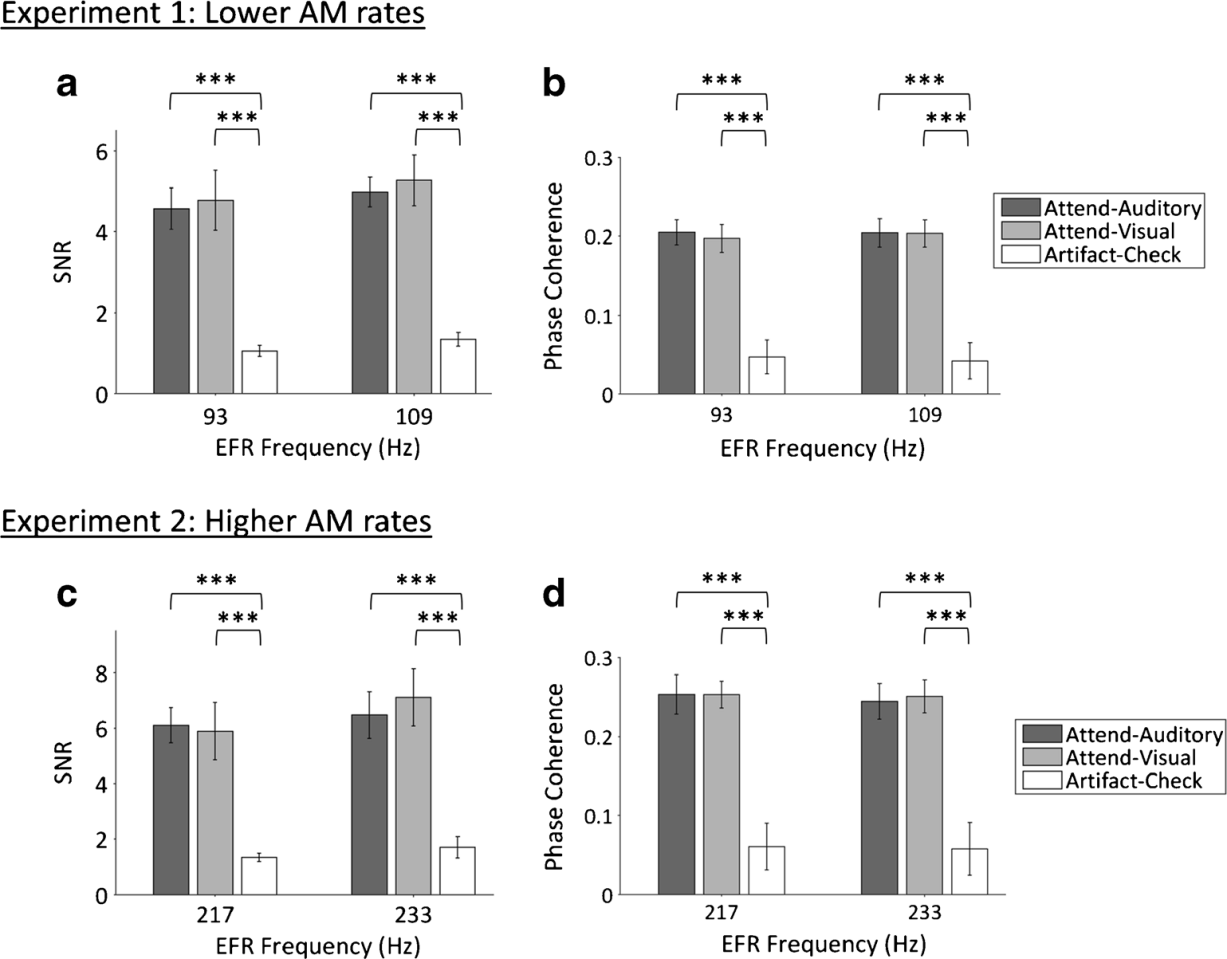
**a** Envelope-following response (EFR) signal-to-noise ratios (SNRs) for Experiment 1 (*N* = 24) at the two EFR components, collapsed across the two different acoustic stimulus types (i.e. low or high stream first). **b** EFR phase coherence in experiment 1 (*N* = 24) at the two EFR components, collapsed across the two different acoustic stimulus types (i.e. low or high stream first). **c**, **d** Same for Experiment 2 (*N* = 12). *AM* amplitude modulation

We used two-tailed within-subject ANOVAs as a first step to compare EFR SNRs and phase coherence across conditions (Attend-Auditory, Attend-Visual, and Artefact Check) and EFR components (93 and 109 Hz). SNRs differed among the Attend-Auditory, Attend-Visual, and Artefact Check conditions [*F*(1.5, 35.3) = 71.0, *p* < 0.001, *⍵*_*p*_^2^ = 0.74]. EFR SNRs were significantly greater in the Attend-Auditory and Attend-Visual conditions than in the Artefact Check condition [*F*(1, 23) = 139.5, *p* < 0.001, *⍵*_*p*_^2^ = 0.85 and *F*(1, 23) = 83.6, *p* < 0.001, *⍵*_*p*_^2^ = 0.77, respectively; see Fig. 3a], meaning that EFRs did not arise due to stimulus artefacts. There was a trend for greater SNRs in the Attend-Visual than Attend-Auditory condition, although the post hoc comparison was not significant after Bonferroni correction (*p* = 0.079). EFR SNRs did not significantly differ between the two EFR components [*F*(1, 23) = 3.49, *p* = 0.08, *⍵*_*p*_^2^ = 0.09]. There was no significant interaction between Condition and EFR component [*F*(2, 46) = 0.60, *p* = 0.56, *⍵*_*p*_^2^ = − 0.02].

Similarly, phase coherence differed among the Attend-Auditory, Attend-Visual, and Artefact Check conditions [*F*(2, 46) = 94.13, *p* < 0.001, *⍵*_*p*_^2^ = 0.79]. Phase coherence in the Attend-Auditory and Attend-Visual conditions were significantly greater than in the Artefact Check condition [*F*(1, 23) = 123.09, *p* < 0.001, *⍵*_*p*_^2^ = 0.83 and *F*(1, 23) = 112.46, *p* < 0.001, *⍵*_*p*_^2^ = 0.82, respectively; see Fig. 3b]. Bonferroni-corrected post hoc tests showed no significant difference in phase coherence between the Attend-Auditory and Attend-Visual conditions (*p* ≈ 1.00). Phase coherence did not differ significantly between 93 and 109 Hz [*F*(1, 23) = 1.71, *p* = 0.20, *⍵*_*p*_^2^ = 0.03], and there was no significant interaction between Condition and EFR component [*F*(2, 46) = 0.14, *p* = 0.87, *⍵*_*p*_^2^ = − 0.04].

#### Frequency-Specific Attention Affects EFR Signal-to-Noise Ratio

Table 1 displays mean EFR SNRs separately for the Attend-High, Attend-Low, and Attend-Visual (low or high stream first) conditions. Paired-sample *t* tests showed that EFR SNRs in the Attend-Visual condition differed significantly between trials in which the low or high stream began first, even though participants’ task was identical in those trials [93 Hz: *t*(23) = 4.22, *p* < 0.001; 109 Hz: *t*(23) = 2.27, *p* = 0.033]. These results suggest that minor differences in the acoustic stimuli between these trials could potentially contribute to differences in EFRs.

**Table 1:** EFR signal-to-noise ratios (calculated as the EFR amplitude at the frequency of interest divided by the average amplitude in the noise bands at adjacent frequencies) in the Attend-Auditory (Attend-High and Attend-Low) and Attend-Visual (high or low stream beginning first) conditions at the high and low EFR components

Condition	Experiment 1		Experiment 2	
	93 Hz	109 Hz	217 Hz	233 Hz
Attend-Low	4.7 ± 1.6	4.8 ± 1.8	6.2 ± 1.8	6.4 ± 2.1
Attend-High	4.4 ± 2.2	5.2 ± 1.5	6.0 ± 2.0	6.5 ± 2.5
Attend-Visual (low stream first)	4.1 ± 2.4	5.6 ± 2.2	6.1 ± 2.1	7.1 ± 2.8
Attend-Visual (high stream first)	5.5 ± 3.1	4.9 ± 1.8	5.7 ± 3.3	7.1 ± 3.2

The Attend-High and Attend-Low conditions presented identical acoustic stimuli as the Attend-Visual (high stream first) and Attend-Visual (low stream first) conditions. Thus, we normalised EFRs in the Attend-High and Attend-Low conditions by the evoked EFRs in the corresponding Attend-Visual condition that contained the identical acoustic stimulus (i.e. high or low stream first, respectively). In the Attend-Auditory conditions, the first tone cued the frequency to be attended, whereas in the Attend-Visual conditions the stream that started first was not relevant for the task.

**Fig. 4:**
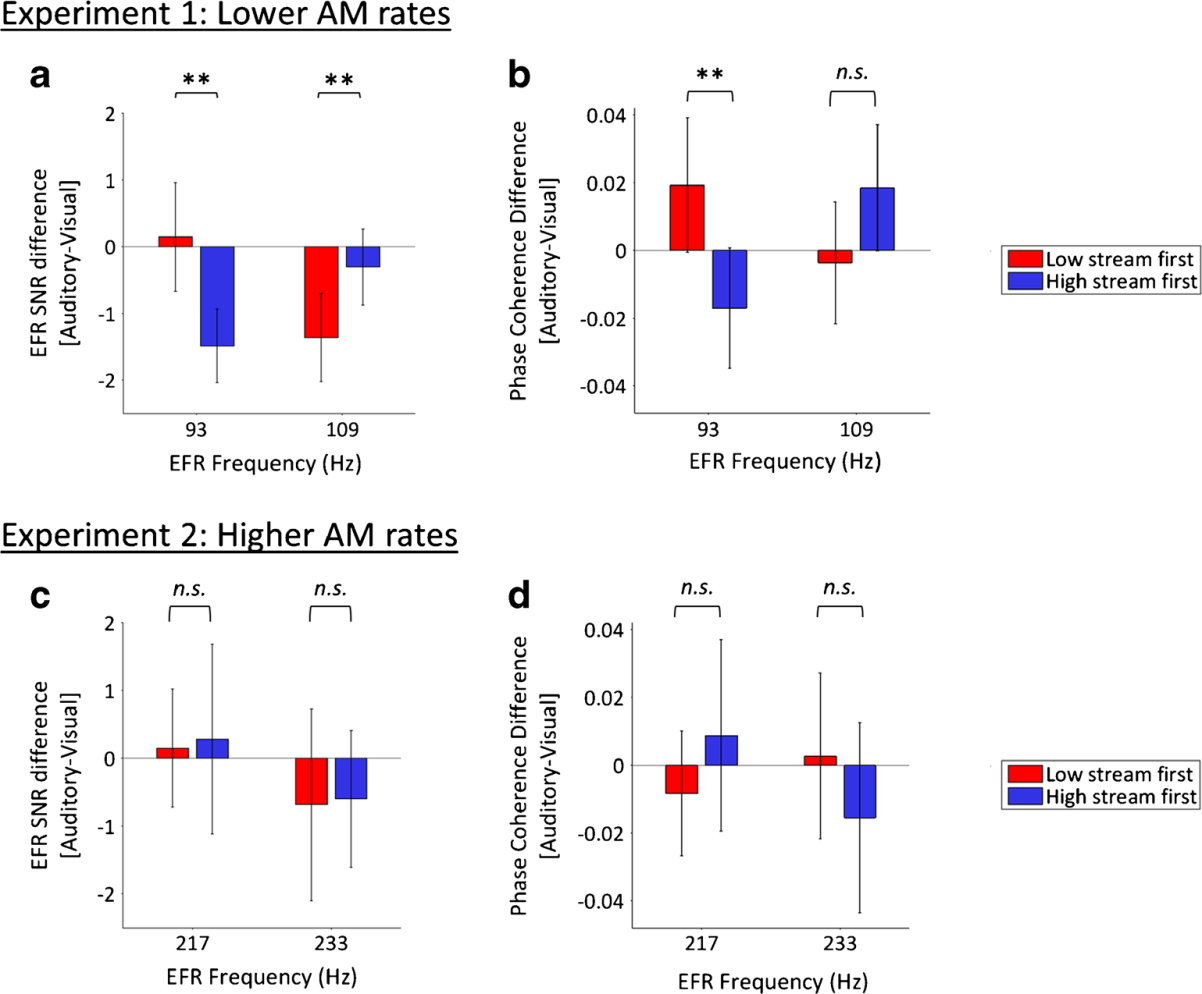
**a** Difference in SNR between the Attend-Auditory and Attend Visual conditions in Experiment 1 (*N* = 24), for trials in which the acoustic stimuli were identical. **b** Difference in phase coherence between the Attend-Auditory and Attend-Visual conditions of experiment 1 (*N* = 24), for trials in which acoustic stimuli were identical. **c**, **d** Same for Experiment 2 (*N* = 12). *AM* amplitude modulation

Figure 4a shows the difference in SNRs between the Attend-Auditory and Attend-Visual conditions, for trials in which the acoustic stimuli were identical. A within-subject two-way ANOVA examining the effect of EFR component (low or high) and Attended frequency (low or high) on these SNR difference values revealed no main effect of EFR component [*F*(1, 23) = 0.18, *p* = 0.68, *⍵*_*p*_^2^ = −0.03] or of Attended frequency [*F*(1, 23) = 1.45, *p* = 0.24, *⍵*_*p*_^2^ = 0.02], but a significant interaction [*F*(1, 23) = 18.81, *p* < 0.001, *⍵*_*p*_^2^ = 0.42].

At the AM rate that tagged the low-frequency carrier (93 Hz), the SNR difference was significantly greater when attention was directed to the low tone stream than the high tone stream [*t*(23) = 3.77, *p* = 0.001, *d*_z._ = 0.77]. The opposite pattern was obtained for the AM rate that tagged the high-frequency carrier (109 Hz): the SNR difference was significantly greater when attention was directed to the high tone stream than the low tone stream [*t*(23) = 2.98, *p* = 0.007, *d*_z_ = 0.61]. These results indicate that frequency-specific attention significantly modulated EFR SNRs at the lower AM rates (93 and 109 Hz) that are typically used in EFR studies.

#### Frequency-Specific Attention Affects EFR Phase Coherence

Next, we analysed phase coherence. Table 2 displays mean EFR phase coherence separately for the Attend-High, Attend-Low, and Attend-Visual (high or low stream first) conditions. Figure 4b illustrates the difference in phase coherence between the Attend-Auditory and Attend-Visual conditions, for trials in which the acoustic stimuli were identical. A within-subject two-way ANOVA examining the effect of EFR component (low or high) and Attended frequency (low or high) on these phase coherence difference values showed no main effect of EFR component [*F*(1, 23) = 0.78, *p* = 0.39, *⍵*_*p*_^2^ = −0.01] or Attended frequency [*F*(1, 23) = 0.36, *p* = 0.56, *⍵*_*p*_^2^ = − 0.03]. However, the two-way interaction between EFR component and Attended frequency was significant [*F*(1, 23) = 12.31, *p* = 0.002, *⍵*_*p*_^2^ = 0.31].

**Table 2:** EFR phase coherence values in the Attend-Auditory (Attend-High and Attend-Low) and Attend-Visual (high or low stream beginning first) conditions at the high and low EFR components

Condition	Experiment 1		Experiment 2	
	93 Hz	109 Hz	217 Hz	233 Hz
Attend-Low	0.20 ± 0.09	0.19 ± 0.10	0.23 ± 0.08	0.22 ± 0.08
Attend-High	0.21 ± 0.07	0.22 ± 0.07	0.28 ± 0.12	0.26 ± 0.12
Attend-Visual (low stream first)	0.18 ± 0.10	0.21 ± 0.10	0.24 ± 0.08	0.22 ± 0.09
Attend-Visual (high stream first)	0.21 ± 0.07	0.20 ± 0.07	0.27 ± 0.11	0.28 ± 0.08

At the AM rate that tagged the low-frequency carrier (93 Hz), the phase coherence difference was significantly greater when attention was directed to the low tone stream than the high tone stream [*t*(23) = 3.06, *p* = 0.005, *d*_z_ = 0.62], demonstrating an effect of frequency-specific attention at the low EFR component. There was a trend towards greater phase coherence at the high EFR component (109 Hz) when attention was directed to the high tone stream than the low tone stream, although the difference was not significant [*t*(23) = 1.98, *p* = 0.060, *d*_z_ = 0.40].

#### No Relationship Between Task Performance and Attentional Modulation of EFRs

**Fig. 5:**
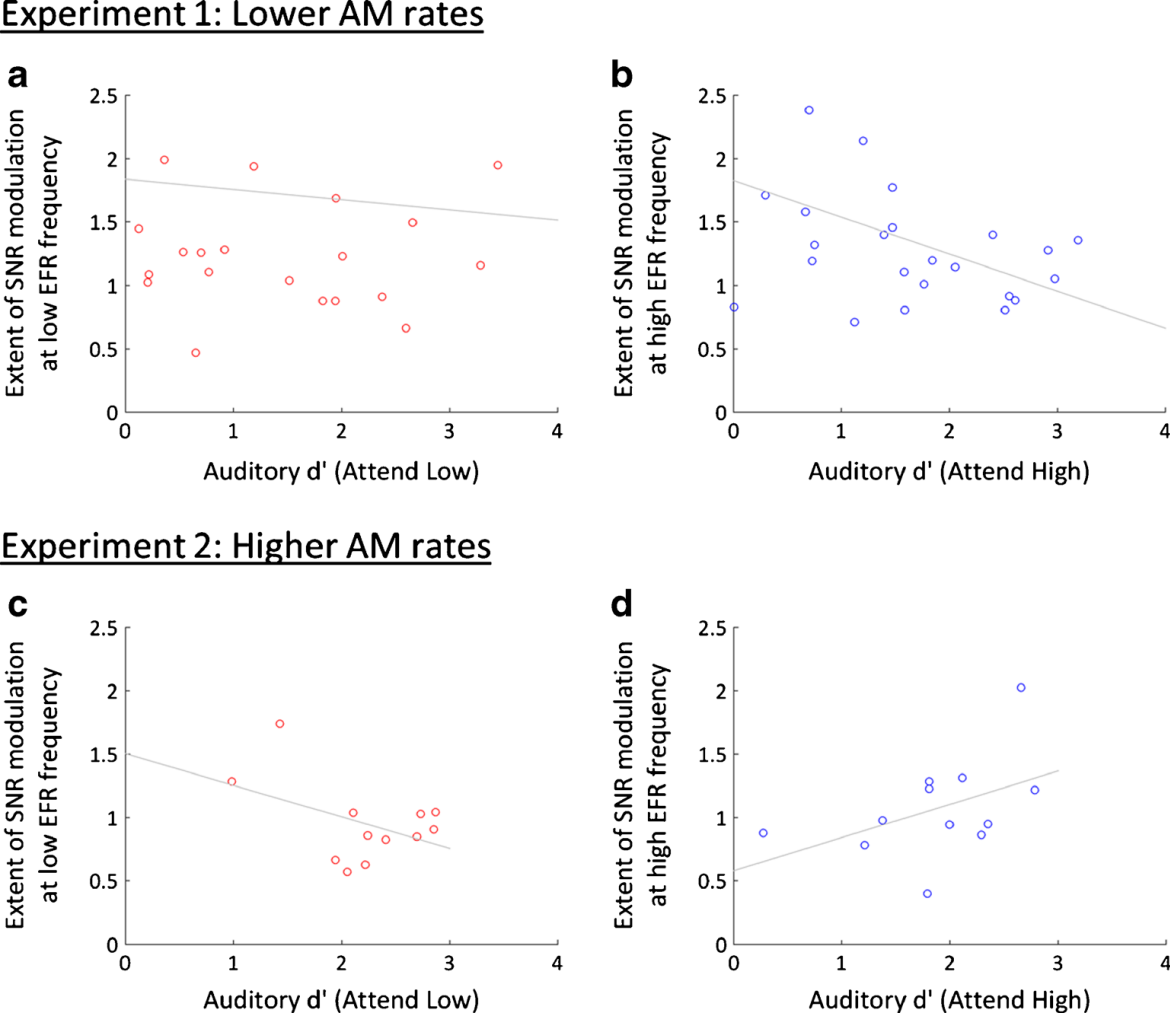
Scatter plots showing no relationship between sensitivity (d′) for the Attend-Auditory task and the extent of EFR SNR modulation by attention. **a** d′ when participants were attending to the low-frequency stream and the extent of attentional SNR modulation at the low EFR component (93 Hz) in experiment 1. Each circle illustrates the results from an individual participant. Least-square lines of best fit are displayed in grey. **b** d′ when participants were attending to the high-frequency stream and the extent of attentional SNR modulation at the high EFR component (109 Hz) in Experiment 1. **c**, **d** Same for Experiment 2 (*N* = 12), for the low (217 Hz) and high (233 Hz) EFR components. *AM* amplitude modulation

There were large individual differences in behavioural performance in the Attend-Auditory task (with some participants responding with low sensitivity). As poor performance could indicate that participants were not deploying frequency-specific attention, we investigated whether only those participants who responded with high sensitivity showed attentional modulation of EFRs. Figure 5a, b displays auditory d′ (in the Attend-Low and Attend-High tasks) and the extent of attentional modulation of EFR SNRs for each participant. Bonferroni-corrected Pearson’s product-moment correlations revealed no relationship between task performance and attentional modulation of SNRs at 93 Hz (*r* = − 0.06, *p* ~ 1.00). At 109 Hz, there was a trend towards a negative correlation (*r* = −0.45) that just missed significance (*p* = 0.054).

Bonferroni-corrected Pearson’s product-moment correlations between auditory d′ and the extent of attentional modulation of phase coherence values revealed no significant relationship at 93 Hz (*r* = − 0.24, *p* ~ 1.00). Although, similar to the SNR results, there was a trend towards a negative correlation at 109 Hz (*r* = − 0.48, *p* = 0.07).

### Experiment 2: High AM Rates

#### Task Performance

Sensitivity (d′) varied among participants in both the auditory (range 1.1–2.7; based on all trials, irrespective of attention condition) and visual (range 1.6–2.6) tasks. There was no significant difference in task performance between the visual (*x̅ =* 2.1, *s* = 0.3) and auditory (*x̅ =* 2.0, *s* = 0.6) tasks [*t*(11) = 1.00, *p* = 0.34], or between Attend-High (*x̅ =* 1.9, *s* = 0.7) and Attend-Low (*x̅ =* 2.2, *s* = 0.6) trials [*t*(11) = 1.93, *p* = 0.08] (Fig. 2b). Participants did not frequently respond to deviants in the non-target stream (Attend-Low: *x̅ =* 1.8 % of non-target deviants, *s* = 1.5; Attend-High: *x̅ =* 2.2 %, *s* = 3.0).

#### Comparison of EFRs Between Conditions and Spectral Components

We confirmed that the EFRs in the Attend-Auditory and Attend-Visual conditions could not be explained by stimulus artefact (see Fig. 3c, d). A within-subject two-way ANOVA (Condition × EFR component) showed a significant difference in SNRs between the Attend-Auditory, Attend-Visual, and Artefact Check conditions [*F*(2, 22) = 86.6, *p* < 0.001, *⍵*_*p*_^2^ = 0.87]. SNRs in the Attend-Auditory and Attend-Visual conditions were significantly greater than in the Artefact Check condition [*F*(1, 11) = 127.9, *p* < 0.001, *⍵*_*p*_^2^ = 0.91 and *F*(1, 11) = 96.2, *p* < 0.001, *⍵*_*p*_^2^ = 0.88, respectively; see Fig. 3c]. EFR SNRs were greater in the Attend-Visual than in the Attend-Auditory condition (*p* = 0.013), due to similar amplitudes (*p* ≈ 1.00) but greater noise in the Attend-Auditory condition (*p* = 0.007). SNRs did not differ significantly between 217 and 233 Hz [*F*(1, 11) = 0.86, *p* = 0.38]. There was also no significant interaction between Condition and EFR component [*F*(2, 22) = 0.30, *p* = 0.75, *⍵*_*p*_^2^ = −0.01].

Phase coherence also differed among the Attend-Auditory, Attend-Visual, and Artefact Check conditions [*F*(2, 22) = 89.19, *p* < 0.001, *⍵*_*p*_^2^ = 0.88]. Phase coherence values in the Attend-Auditory and Attend-Visual conditions were significantly greater than in the Artefact Check condition [*F*(1, 11) = 91.32, *p* < 0.001, *⍵*_*p*_^2^ = 0.87 and *F*(1, 11) = 114.46, *p* < 0.001, *⍵*_*p*_^2^ = 0.90, respectively; see Fig. 3d]. Bonferroni-corrected post hoc tests showed no significant difference in phase coherence between the Attend-Auditory and Attend-Visual conditions (*p* ≈ 1.00). Phase coherence did not differ significantly between 217 and 233 Hz [*F*(1, 11) = 2.21, *p* = 0.17, *⍵*_*p*_^2^ = 0.09], and there was no significant interaction between Condition and EFR component [*F*(2, 22) = 0.83, *p* = 0.45, *⍵*_*p*_^2^ = 0.01].

#### Similar Magnitude EFRs as Experiment 1

We checked whether we were measuring comparable EFRs in the Attend-Auditory and Attend-Visual conditions of Experiment 2 as in Experiment 1. There was no significant difference in phase coherence between the experiments (Experiment 1: *x̅ =* 0.20, *s* = 0.07; Experiment 2: *x̅ =* 0.25, *s* = 0.07) [*t*(34) = 1.89, *p* = 0.07, *g*_s_ = 0.65]. However, overall, EFR SNRs were significantly greater in Experiment 2 (*x̅ =* 6.40, *s* = 1.74) than in Experiment 1 (*x̅ =* 4.90, *s* = 1.74) [*t*(34) = 2.43, *p* = 0.010, *g*_s_ = 0.84]. Thus, we recorded sufficiently robust EFRs to detect attentional modulations of EFRs in Experiment 2 with at least as high power as in Experiment 1.

#### No Effect of Frequency-Specific Attention on EFR Signal-to-Noise Ratio

Table 1 displays mean EFR SNRs separately for the Attend-High, Attend-Low, and Attend-Visual (low or high stream first) conditions. Figure 4c illustrates the difference in SNRs between the Attend-Auditory and Attend-Visual conditions at the two EFR components for trials in which the acoustic stimuli were identical. A within-subject two-way ANOVA examining the effect of EFR component (low or high) and Attended frequency (low or high) on these SNR difference values revealed no main effect of EFR component [*F*(1, 11) = 1.65, *p* = 0.22, *⍵*_*p*_^2^ = 0.05] or Attended frequency [*F*(1, 11) = 0.04, *p* = 0.84, *⍵*_*p*_^2^ = −0.08]. The two-way interaction between EFR component and Attended frequency was not significant either [*F*(1, 11) < 0.01, *p* = 0.95, *⍵*_*p*_^2^ = − 0.08]. Thus, frequency-specific attention had no influence on EFRs at the AM rates used in Experiment 2.

#### No Effect of Frequency-Specific Attention on EFR Phase Coherence

Next, we analysed phase coherence values. Table 2 displays mean EFR phase coherence separately for the Attend-High, Attend-Low, and Attend-Visual (low or high stream first) conditions. Figure 4d illustrates the difference in phase coherence between the Attend-Auditory and Attend-Visual conditions at the two EFR components, for trials in which the acoustic stimuli were identical. A within-subject two-way ANOVA examining the effect of EFR component (low or high) and Attended frequency (low or high) on these phase coherence difference values revealed no main effect of EFR component [*F*(1, 11) = 0.46, *p* = 0.51, *⍵*_*p*_^2^ = − 0.04] or Attended frequency [*F*(1, 11) < 0.01, *p* = 0.96, *⍵*_*p*_^2^ = − 0.08]. The two-way interaction between EFR component and Attended frequency was not significant either [*F*(1, 11) = 1.68, *p* = 0.22, *⍵*_*p*_^2^ = 0.05].

#### No Relationship Between Task Performance and Attentional Modulation of EFRs

Figure 5c, d displays auditory d′ and the extent of attentional EFR SNR modulation for each participant at the two EFR components. Bonferroni-corrected Pearson’s product-moment correlations revealed no relationship between behavioural performance on Attend-Low trials and attentional modulation at the low EFR component (*r* = −0.45, *p* = 0.29) or between behavioural performance on Attend-High trials and attentional modulation at the high EFR component (*r* = 0.46, *p* = 0.27). Bonferroni-corrected Pearson’s product-moment correlations between auditory d′ and the extent of attentional modulation of phase coherence values revealed no significant relationship at the low EFR component (*r* = − 0.001, *p* ~ 1.00) or the high EFR component (*r* = − 0.20, *p* ~ 1.00).

### Comparison of Experiments 1 and 2

We found frequency-specific attentional modulation of EFR SNRs and phase coherence in Experiment 1, but not in Experiment 2. To identify whether the differences between experiments were robust, we conducted two mixed three-way ANOVAs—separately for SNRs and phase coherence—with within-subject factors of EFR component and Attended frequency and a between-subject factor of Experiment.

There was a significant three-way interaction of Experiment, EFR component, and Attended frequency for SNRs [*F*(1, 34) = 6.95, *p* = 0.013, *⍵*_*p*_^2^ = 0.14]. There was also a significant three-way interaction for phase coherence [*F*(1, 34) = 9.51, *p* = 0.004, *⍵*_*p*_^2^ = 0.19]. These results indicate that the patterns of results indeed differed significantly between the two experiments.

Next, we tested whether differences in behavioural performance could be responsible for different results between the experiments. Performance (d′) on the auditory task did not differ significantly between experiments [*t*(34) = 2.00, *p* = 0.054, *g*_s_ = 0.69], nor did performance on the visual task [*t*(34) = 0.55, *p* = 0.59, *g*_s_ = 0.19].

## Discussion

We found frequency-specific attentional modulation of EFRs at lower (93 and 109 Hz) but not at higher (217 and 233 Hz) stimulus AM rates. At lower rates (Experiment 1), EFRs were larger and showed stronger phase coherence when listeners were attending to the tone stream (low- or high-frequency carrier) that was tagged with that AM rate, compared to when they were attending to the other tone stream. However, at higher AM rates (Experiment 2), we found no effect of frequency-specific attention on EFRs, even though other procedures were identical and behavioural performance, EFR SNRs, and EFR phase coherence values were as good as or better than in Experiment 1. If frequency-specific attention modulated *brainstem* components of EFRs (in contrast to cortical components), attentional modulation of EFRs should have been present for both the lower and higher ranges of AM rates.

The current experiments are the first to examine attentional modulation of EFRs at two distinct sets of frequencies and using two complementary measures of EFR magnitude. Within each of our two experiments, we incorporated a replication, demonstrating the same pattern of results at two different EFR components (i.e. corresponding to the higher and lower AM rates) and with two different EFR measures (i.e. SNRs and phase coherence). In Experiment 1, EFR SNRs and phase coherence were modulated by attention at both 93 and 109 Hz (although for phase coherence at 109 Hz the trend was not significant). In Experiment 2, we found no evidence of attentional modulation of SNRs or phase coherence at either 217 or 233 Hz. Importantly, we provide strong evidence for a dissociation between the two ranges of AM rates—the patterns of results differed statistically between the two experiments.

The fact that we observed attentional modulation for frequencies with suspected cortical contributions, but not at frequencies higher than the cortex is thought to be capable of tracking, suggests that attentional modulation of EFRs at lower AM rates could result from attentional modulation of a cortical component contributing to the measured EFRs. Cortical contributions to frequency-specific attention could not be measured directly in the current experiments. This is because we designed the stimuli to measure phase-locked responses at frequencies with putative brainstem generators and, thus, the stimuli were amplitude modulated at those frequencies. In addition, we presented sequences of repeated, long-duration tones, meaning that components in filtered time-domain averages were not readily identifiable due to neural adaptation (Sams et al. 1993; Herrmann et al. 2014). However, the results provide strong evidence that different neural processes underlie activity at the higher and lower frequencies tested. Based on evidence from ECoG showing cortical frequency-following in Heschl’s gyrus up to but not beyond 200 Hz (Brugge et al. 2009; Nourski et al. 2013; Behroozmand et al. 2016) and recent MEG (Coffey et al. 2016) and EEG (Coffey et al. 2017) studies showing that the generators of the frequency-following response at 98 Hz most likely include cortex, we suspect that the observed dissociation might arise from cortical contributions to EFRs at the lower frequencies we tested in Experiment 1 and not at the higher frequencies we tested in Experiment 2. Although less likely, another possibility is that different findings at higher- and lower-modulation frequencies reflect the contribution of different combinations of brainstem generators to EFRs (see Marsh et al. 1974; Dykstra et al. 2016). The current results add to the growing literature by demonstrating that the most popular method for recording EFRs—EEG—is sensitive to different neural processes at frequencies above and below 200 Hz, within the range of frequencies at which EFRs are typically assumed to reflect brainstem processes. Furthermore, we show that this difference has the potential to dramatically alter the conclusions of EFR studies.

The results of Experiment 1 show that EFRs elicited by an AM tone have greater SNRs when that tone is attended or when visual stimuli are attended than when attention is directed to a different-frequency tone (Fig. 4a). This result suggests that attention suppresses the amplitude of EFRs to tones at frequencies that are not attended. The results also show that EFRs elicited by an AM tone have greater phase coherence when that tone is attended than when attention is directed to a different-frequency tone or to visual stimuli, suggesting that attention enhances EFR phase coherence for tones at attended frequencies (Fig. 4b). Suppression of EFR amplitudes to an unattended tone was also reported by Hairston et al. (2013). They measured following responses to the temporal fine structure of a ‘background’ 220-Hz pure tone. Participants performed either a temporal discrimination task on pure tones with a frequency of 587 Hz, a visual temporal discrimination task, or no task. The amplitude of the response was lower during the auditory than the visual and no-task conditions. The current results are consistent with those reported by Hairston et al. (2013).

Unlike Experiment 1, two previous studies found no consistent modulation of EFRs by auditory attention (Lehmann and Schönwiesner 2014; Varghese et al. 2015)—although those experiments cued attention to spoken words at different spatial locations (which also differed in fundamental frequency), rather than explicitly to sounds of different frequencies. Varghese et al. (2015) analysed EFRs at similar frequencies (97 and 113 Hz) as the modulation frequencies employed in Experiment 1, but they obtained much poorer SNRs—perhaps attributable to a shorter analysis window and fewer epochs, which likely reduced their ability to detect significant attentional modulation. Lehmann and Schönwiesner (2014) report high SNRs, but used stimuli with relatively high fundamental frequencies of 170 and 225 Hz. They observed attentional modulation in the expected direction at 170 Hz (with dichotic presentation) but not at 225 Hz, which is similar to the higher AM rates used in Experiment 2. The results of the current experiments add, crucially, to the ongoing debate of whether attention affects EFRs by showing that choice of modulation rate can affect the outcomes of EFR studies, which could potentially reconcile seemingly disparate results found in previous studies. The findings of Lehmann and Schönwiesner (2014) are consistent with the results reported here, which reveal attentional modulation at frequencies below 200 Hz (Experiment 1), but not at those above 200 Hz (Experiment 2).

Galbraith and Doan (1995) did find attentional modulation at 400 Hz, which is of higher frequency than the cortex is assumed capable of tracking. However, they cued spatial attention to the left or right ear and recorded following responses to the temporal fine structure, instead of the envelope. Thus, it is possible that frequency-specific attentional modulation of brainstem responses in EEG is more difficult to detect than attention shifts between ears and/or that temporal fine structure following responses reflect different neural processes than envelope responses.

We found no difference in EFR amplitudes and phase coherence between the Attend-Auditory and Attend-Visual conditions overall. Although some previous studies reported modulation of EFRs by visual or auditory attention, the findings are inconsistent: some studies found a difference in amplitudes, but not latencies (e.g. Galbraith and Arroyo 1993; Galbraith et al. 2003), others found a difference in latencies but not amplitudes (e.g. Hoormann et al. 2000), and some reported no differences (e.g. Galbraith and Kane 1993; Varghese et al. 2015). The current finding is not surprising in the context of these previous results. Given that d′ in the current experiment was approximately 2, participants were performing the visual task accurately, making it unlikely that the visual task used in the current experiments did not effectively engage attention.

In Experiment 1, there was a difference in EFR SNRs and phase coherence between the two stimulus types (low or high stream first) in the Attend-Visual condition. We expected to observe no difference in EFRs between these trials relating to attention because the stimulus that began first was irrelevant to the visual task. There are several possible explanations for this finding, which cannot be distinguished here. First, differences in EFRs may reflect acoustic differences between the two stimulus types (i.e. low or high stream first). Second, it is possible that attention did in fact differ between the two stimulus types in the Attend-Visual condition: the onset of the tone streams could have captured attention exogenously. One attention-driven explanation is that each stream captured attention sequentially; thus, the stream that began last in the Attend-Visual condition would capture attention throughout the analysis window, meaning that the stream that began first would be unattended—potentially causing lower EFR SNRs and phase coherence at the AM rate of the first tone stream. A different attention-driven explanation is that the tone stream that began first may have been most salient; if listeners actively suppressed the percept of the stream that began first to help them focus on the visual task, then EFR SNRs and phase coherence would again be lower at the AM rate of the first tone stream. The stimulus-driven and attention-driven explanations could be distinguished in future studies by presenting acoustically identical stimuli in Attend-Low and Attend-High trials and by using a visual, rather than acoustic, stimulus to cue attention. The two attention-driven explanations could be distinguished by analysing EFRs based on the order of streams; if participants suppressed the tone stream that began first, the order of the two later streams should not affect EFRs.

Our results demonstrate that measuring EFRs at different frequencies within the range of frequencies that are typically assumed to reflect brainstem processing has the potential to dramatically alter the conclusions of EFR studies. If we had only measured EFRs at the lower frequencies used in Experiment 1, we may have concluded that attention influences brainstem encoding, whereas, if we had only used the higher frequencies of Experiment 2, we may have concluded that there is no influence of attention on brainstem encoding. Thus, our findings have important implications for experiments comparing EFRs across different populations. Previous studies have found that EFRs elicited by musical notes differ between musicians and non-musicians (Musacchia et al. 2007), EFRs elicited by Mandarin sounds differ between speakers of Mandarin and English (Krishnan et al. 2009, 2010), and EFRs elicited by spoken syllables differ between children with different speech-in-noise abilities (Anderson et al. 2010). Those results have been attributed to differences in brainstem encoding. However, given that EFRs are typically recorded at lower frequencies (70–200 Hz), the reported differences in EFRs could potentially arise from differences in *cortical* attentional processes rather than brainstem processes. We suggest that the findings of these studies should be re-evaluated and recommend further work aimed at disambiguating cortical and brainstem responses. For example, future studies could present stimuli with fundamental frequencies above 200 Hz to confidently attribute EFRs to brainstem generators. Also, different methods could be used to more clearly separate brainstem responses and cortical activity (e.g. MEG or functional magnetic resonance imaging [fMRI], albeit at the cost of losing information about phase locking). In particular, fMRI, with its very high spatial resolution, might be a promising method to evaluate attentional and cognitive modulation of auditory brainstem (inferior colliculus) and thalamus (medial geniculate body) activity as fMRI has previously been used to show modulation of brainstem responses by spatial attention (Rinne et al. 2008).

## Conclusions

Using EEG—currently the most common method for recording EFRs—we found that frequency-specific attention affected the amplitude of EFRs elicited by stimuli with amplitude modulation rates of 93 and 109 Hz, but not by stimuli with amplitude modulation rates of 217 and 233 Hz. The effect of attention was significantly stronger at the lower two modulation rates than at the higher two rates. We conclude that EFRs at lower amplitude modulation rates reflect different processes (e.g. a cortical contribution, which is modulated by attention) than EFRs above 200 Hz. The significant difference in results between the two sets of AM rates demonstrates that EEG-recorded EFRs reflect different processes for AM rates below 200 Hz (which are commonly used in EFR research) compared to higher rates. Critically, this finding should lead to re-evaluation of previous studies claiming that differences in EFRs reflect differences in brainstem encoding.
